# Bicaudal-D Regulates Fragile X Mental Retardation Protein Levels, Motility, and Function during Neuronal Morphogenesis

**DOI:** 10.1016/j.cub.2010.07.016

**Published:** 2010-08-24

**Authors:** Ambra Bianco, Martin Dienstbier, Hannah K. Salter, Graziana Gatto, Simon L. Bullock

**Affiliations:** 1 Cell Biology Division, MRC Laboratory of Molecular Biology, Hills Road, Cambridge CB2 0QH, UK

**Keywords:** MOLNEURO, CELLBIO, DEVBIO

## Abstract

The expression of the RNA-binding factor Fragile X mental retardation protein (FMRP) is disrupted in the most common inherited form of cognitive deficiency in humans. FMRP controls neuronal morphogenesis by mediating the translational regulation and localization of a large number of mRNA targets [1–3], and these functions are closely associated with transport of FMRP complexes within neurites by microtubule-based motors [2–4]. However, the mechanisms that link FMRP to motors and regulate its transport are poorly understood. Here we show that FMRP is complexed with Bicaudal-D (BicD) through a domain in the latter protein that mediates linkage of cargoes with the minus-end-directed motor dynein. We demonstrate in *Drosophila* that the motility and, surprisingly, levels of FMRP protein are dramatically reduced in *BicD* mutant neurons, leading to a paucity of FMRP within processes. We also provide functional evidence that BicD and FMRP cooperate to control dendritic morphogenesis in the larval nervous system. Our findings open new perspectives for understanding localized mRNA functions in neurons.

## Graphical abstract

**Graphical Abstract:**

**Figure:**
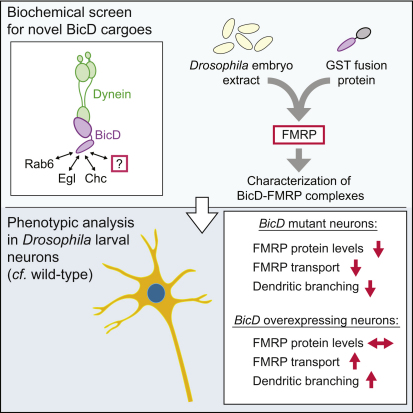

**Highlights:**

► FMRP is a novel interactor of the dynein cofactor BicD ► BicD is required for motility and normal levels of FMRP in neurons ► Long-distance, bidirectional transport mediates spreading of FMRP into neuritis ► BicD and FMRP cooperate to control morphogenesis of neuronal dendrites

## Results and Discussion

BicD proteins (BicD in *Drosophila* and BicD1 and BicD2 in mammals) play roles in the transport of a subset of cargoes by the minus-end-directed microtubule motor dynein. The N-terminal two-thirds of BicD interact with dynein and its accessory complex dynactin, and the C-terminal third (the C-terminal domain [CTD]) mediates mutually exclusive association with different cargoes [5–8]. The best-characterized roles of BicD proteins are in the bidirectional transport of Golgi vesicles and a subset of asymmetrically localized *Drosophila* mRNAs, which are mediated by binding of the CTD to the membrane-associated G protein Rab6 [9] and the RNA-binding protein Egalitarian (Egl) [7], respectively. The interactions of the BicD CTD with both proteins are inhibited by the K730M substitution in the *Drosophila* BicD protein [7], which is a null mutation in vivo [10]. K730M does not, however, inhibit binding of the BicD CTD to other copies of BicD [7], indicating that it specifically effects association of BicD with motor cargoes.

### The BicD CTD Recruits FMRP

**Figure 1:**
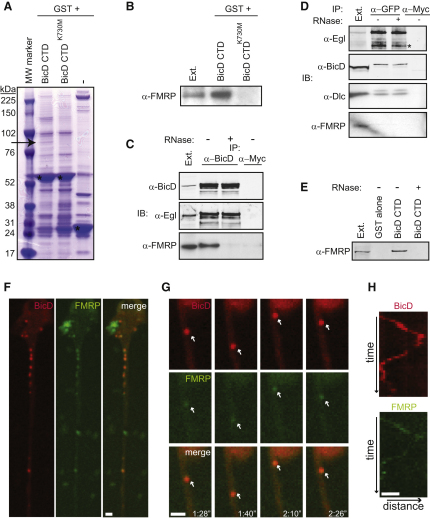
FMRP Is a Novel BicD-Associated Protein and Colocalizes with BicD in Moving Particles within Neurons (A and B) Pull-down from embryo extracts via the wild-type or K730M BicD CTD fused to GST. (A) Coomassie stain; arrow indicates the FMRP band enriched on the wild-type CTD and asterisks mark GST fusion proteins. (B) The identity of FMRP was confirmed by western blotting. (C) Endogenous FMRP specifically coprecipitates with endogenous BicD from *Drosophila* embryonic extracts in an RNase-sensitive manner. BicD:Egl complexes are insensitive to RNase. IP, immunoprecipating antibody; IB, immunoblotting antibody. (D) Unlike the known Egl-interacting proteins BicD and Dynein light chain (Dlc) [28], FMRP is not immunoprecipitated with Egl::GFP. Asterisk marks endogenous Egl that is immunoprecipitated with Egl::GFP in an RNase-independent manner. (E) The pull-down of FMRP from extracts by BicD-CTD is RNase sensitive. Load is 1% of input in (A) to (E). (F) FMRP::GFP and BicD::mCherry colocalize in puncta in live, primary *Drosophila* neurons. See Movie S1A for time-lapse. Puncta have maximum instantaneous velocities of ∼0.7 μm/s, consistent with the involvement of molecular motors. The mean velocity of these motile particles was ∼0.2 μm/s, which is similar to that reported for transported mRNPs in other neuronal cell types [15, 16]. (G) Stills of Movie S1B showing a bidirectional cargo containing FMRP::GFP and BicD::mCherry (arrow). (H) Kymographs of the particle in (G). Left to right in the kymographs represents movement away from the cell body. Bars represent 2 μm. A red and green image set was captured every 2 s.

In an attempt to elucidate the basis of linkage of other cargoes to dynein, we performed a GST pull-down from fly embryonic extracts with the *Drosophila* BicD CTD (amino acids 536–782) and an equivalent K730M mutant protein as a specificity control. Mass spectrometry revealed that a protein of 80–85 kDa reproducibly recruited only by the wild-type CTD (Figure 1A) was *Drosophila* FMRP (27 unique peptides), and this was confirmed by western blotting (Figure 1B). Endogenous BicD and FMRP were specifically coimmunoprecipitated from *Drosophila* embryonic extracts (Figure 1C). Unlike known Egl-interacting proteins, FMRP was not coimmunoprecipitated with a GFP-tagged Egl protein (Figure 1D). This finding, together with the observation that binding of both Egl [7] and FMRP (Figures 1A and 1B) to BicD is impaired by the K730M mutation, suggests that BicD:FMRP complexes are largely, or completely, distinct from BicD:Egl complexes.

The ability to detect FMRP in CTD pull-downs and BicD immunoprecipitations from extracts was abolished by treatment with RNase (Figures 1C and 1E). In contrast, the complex of Egl with BicD was not sensitive to RNase treatment (Figure 1C). Thus, the stable association of BicD and FMRP in extracts is dependent on RNA. Nonetheless, we found a weak interaction of the BicD CTD with a subfragment of FMRP (aa 220–618) in yeast two-hybrid assays (Figure S1A and Table S1 available online). This interaction was specific, as shown by the fact that it was disrupted by the K730M mutation within the BicD CTD (Figure S1A and Table S1). These findings raise the possibility of a direct contact of BicD and FMRP in vivo that is stabilized by the association of FMRP with RNA targets and possibly other RNA-associated proteins.

### FMRP and BicD Are Cotransported in Bidirectional Complexes in Neurons

The above results suggest that BicD could be a functional interactor of FMRP in vivo. We therefore focused our subsequent studies on neurons, where FMRP plays a prominent role. As previously observed [3, 11], endogenous FMRP is enriched in puncta within the cell body and neurites of *Drosophila* primary neurons cultured from larval brains (Figure S1B). Endogenous BicD was also found in puncta in these cells, but these were much more frequent than those containing FMRP (Figure S1B). Although there was overlap of a subset of FMRP puncta with BicD puncta (Figures S1C and S1D), the widespread distribution of BicD precluded a meaningful interpretation about the extent of complex formation of BicD and FMRP in fixed primary neurons (see Figure S1B legend for discussion).

**Figure 2:**
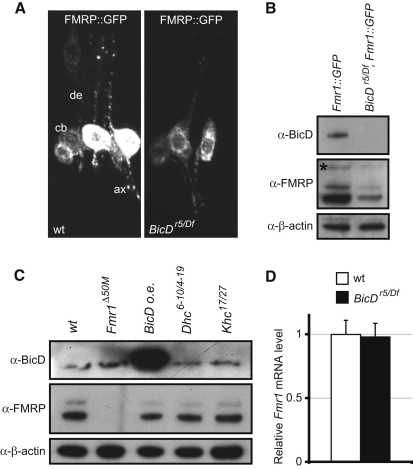
BicD Is Required for FMRP Accumulation in Neuronal Processes and to Maintain FMRP Protein, but Not RNA, Levels (A) Appearance of FMRP::GFP, expressed with the panneuronal driver C155-GAL4, within the chordotonal organ neuron cluster of wild-type and *BicD* mutant third instar larvae (de, dendrites; ax, axons; cb, cell bodies). Images are projections of four z-sections of ∼1 μm each. See Supplemental Experimental Procedures for details of *BicD* null genotype. (B) Western blot showing strongly reduced levels of endogenous FMRP and FMRP::GFP (asterisk) proteins in extracts from *BicD* mutant third instar brains, lacking detectable BicD protein. β-actin acts as a loading control. A similar reduction in endogenous FMRP levels is also observed in the absence of FMRP::GFP (Figure S3A). The intensity of the signal of FMRP in *BicD* mutant extracts was 36% ± 11.5% of the wild-type (mean ± SEM, n = 3; measured from the major endogenous isoform). (C) FMRP levels are unchanged by partial loss-of-function mutations in dynein and kinesin-1 heavy chains, when BicD is strongly overexpressed (o.e.) with C155-GAL4 or in *Fmr1* null mutants. (D) *Fmr1* mRNA levels, normalized to a *β-actin* mRNA control, are not significantly different in wild-type and *BicD* mutant third instar larval brains. For each genotype, n = 3 independent quantitative RT-PCR experiments (each done in duplicate). Error bars represent standard error of the mean (SEM). WT represents wild-type in this and all other figures.

We therefore established neuronal cultures from brains of transgenic larvae expressing FMRP::GFP and BicD::mCherry and used time-lapse microscopy to assay for cotransport of puncta containing both proteins (Figures 1F–1H; Movie S1A and Movie S1B). These fluorescent fusion proteins retain function (Figure S2 and Supplemental Experimental Procedures) and account for ∼20% and 50% of the levels of total FMRP and BicD proteins, respectively, in transgenic larval brain extracts (Figure 2B; data not shown).

Both BicD::mCherry and FMRP::GFP were widely distributed in the cytoplasm of the primary neurons, but bidirectionally transported FMRP::GFP puncta were found in all cells and 92.4% ± 3.2% of them were cotransported with a puncta of BicD:mCherry (mean ± SEM, 135 particles in 20 cells) (Figures 1F–1H; Movie S1A). Thus, FMRP and BicD can be contained within the same motile transport complexes in neurons. The motility of FMRP::GFP in these experiments will be described in more detail below. Only 77.2% ± 4.6% of motile BicD::mCherry puncta were cotransported with a puncta of FMRP::GFP (155 particles in 20 cells), indicating that BicD may transport additional cargoes in these cells and/or that a subset of BicD::mCherry complexes may contain only nonfluorescent, endogenous FMRP.

### BicD Controls FMRP Protein Levels

We next explored whether BicD has a functional role in FMRP:motor complexes in neurons by assessing the subcellular localization of FMRP in third instar *BicD* mutant larvae. Because the high expression of FMRP expression in neighboring nonneuronal cells obfuscates the distribution of the endogenous protein in thin neuronal processes [12], we expressed UAS-FMRP::GFP [12] at low levels by using a panneuronal GAL4 driver. In neurons of zygotic *BicD* null mutant larvae, which also lack detectable maternal BicD protein (Figure 2B), the amount of FMRP::GFP within the neurites was greatly reduced compared to wild-type (Figure 2A). Surprisingly, there was also a much weaker FMRP::GFP signal in the cell body of *BicD* mutant neurons relative to wild-type. Western blotting of third instar larval brain extracts confirmed a striking reduction in levels of both FMRP::GFP and endogenous FMRP in the absence of BicD (Figure 2B; Figure S3A). Strong mutations in genes encoding the dynein and kinesin-1 motor proteins, which should inhibit microtubule-based FMRP transport in *Drosophila* [13], did not alter the amount of FMRP (Figure 2C). These findings, together with observations from interfering with dynactin function (see below), suggest that the reduction in FMRP protein levels in *BicD* mutants is caused by a specific role of BicD rather than an indirect consequence of inefficient FMRP transport.

Levels of the *Fmr1* mRNA, which encodes FMRP, were indistinguishable in wild-type and *BicD* mutant brain extracts, as revealed by quantitative RT-PCR (Figure 2D). Thus, the requirement for BicD in maintaining FMRP protein levels is not associated with RNA decay or transcription. Further evidence against a defect in *Fmr1* transcription in *BicD* mutants is provided by the strong reduction in the levels of the GFP-tagged FMRP protein (Figure 2B), which is transcribed under the control of yeast-derived UAS promoter elements. The FMRP::GFP transgene also lacks the untranslated sequences from the *Fmr1* gene ([12] and data not shown), revealing that BicD's regulation of FMRP protein amount is mediated through the *Fmr1* coding sequence. BicD may therefore influence FMRP protein stability through an unknown mechanism. However, we currently cannot rule out that BicD regulates the translation of FMRP; at least in mammals, the coding sequence of *Fmr1* mRNA contains a translational control element, which negatively regulates protein production by binding FMRP [14]. Distinguishing between these and other possibilities will require long-term studies. Interestingly, the underlying mechanism appears to be restricted to certain cell types as shown by the fact that FMRP levels in cultured *Drosophila* D-Mel cells (a derivative of S2 cells) were not reduced by RNAi-mediated depletion of BicD (Figure S3B).

### BicD Promotes FMRP Motility in Neurons

**Figure 3:**
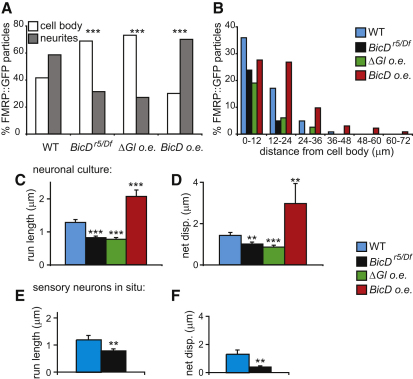
BicD Regulates FMRP Motility within Neurons in Culture and In Situ (A) The proportion of FMRP::GFP particles within neurites of primary cultured neurons is reduced in *BicD* mutants and increased by 2-fold overexpression (o.e.) of BicD (via tub-BicD::mCherry). Panneuronal overexpression (via C155-GAL4) of a dominant-negative dynactin subunit, ΔGlued (ΔGl), also decreases the proportion of FMRP::GFP particles in neurites. (B) The accumulation of FMRP::GFP in distal regions of processes of cultured neurons is similarly sensitive to BicD dosage and ΔGl. *y* axis is percentage of total FMRP::GFP particles in the neuron (i.e., including the cell body). n = 200–393 in (A) and (B). (C–F) Mean values of run length and net displacement (disp.) for only the motile subset of FMRP::GFP particles in primary cultured neurons (C, D; n = 125–182) and chordotonal organs in situ (E, F; n = 25 and 27 in WT and *BicD* mutant, respectively). Run lengths are defined as the distance of travel between reversals or pauses. The frame rate was between 1 and 1.4 s^−1^ for neurons in culture and 2 s^−1^ for larvae. Error bars represent SEM; ^∗∗∗^p < 0.001; ^∗∗^p < 0.01. t tests were used for statistical evaluations (compared to WT), except in (A) (Fisher's exact test using raw numbers).

To investigate whether BicD also has a role in controlling FMRP motility, we examined the distribution of residual FMRP::GFP in *BicD* mutant primary cultured larval neurons. There was a strong decrease in the proportion of FMRP::GFP particles that localized to neurites in *BicD* mutants compared to wild-type (Figure 3A), with FMRP particles also less likely to reach the most distal regions of the mutant processes (Figure 3B). The changes in FMRP distribution are unlikely to result from differences in cellular morphology or general effects on trafficking processes because the length and complexity of neurites, as well as the distribution of mitochondria, was comparable in *BicD* mutant and wild-type neurons (Figures S4A–S4C).

To test directly whether BicD is required for FMRP motility, we performed time-lapse imaging of FMRP::GFP particles in cultured larval neurons. As previously observed [3], and consistent with the dynamics of other mRNP components in neurons [15, 16], FMRP particles in wild-type neurons were usually stationary during several minutes of filming, but some occasionally underwent periods of rapid, directed movement (Movie S2A). Motile particles in the processes exhibited persistent motion both toward and away from the cell body, with some particles rapidly switching directions. There was no overall bias in the length of directed, continuous movements (run lengths) toward and away from the cell body (Figure S4D), consistent with a completely mixed microtubule polarity in both neurites and the soma (Figure S4E).

Bidirectional motion of a subset of FMRP::GFP particles was also observed in primary cultures established from *BicD* mutant larvae (Movie S2A). However, mean run lengths of motile FMRP particles were ∼40% shorter in *BicD* mutant neurons compared to in wild-type neurons (Figure 3C). The net displacement of motile particles was also significantly reduced in mutant neurons (Figure 3D), presumably reflecting a role for long-distance unbiased motor transport in facilitating spreading of cargoes in a process akin to one-dimensional diffusion [17]. In order to investigate the role of BicD in a more physiological context, we filmed chordotonal organ neurons in filleted preparations of third instar larvae. There were similar reductions of FMRP::GFP motility in these neurons in *BicD* mutants, relative to wild-type controls, to those observed in primary cultures (Figures 3E and 3F; Movie S3). Thus, motile FMRP particles require BicD to move efficiently over long distances in neurons both in culture and in situ.

Consistent with BicD's well-characterized role in dynein/dynactin-mediated transport, inhibition of dynactin function by neuron-specific expression of a dominant-negative version of the p150^Glued^ subunit (ΔGlued) [18] strongly reduced the motility of FMRP puncta and their localization into neuronal processes (Figures 3B–3D). Despite the strong difference in efficiency of FMRP transport, the amounts of FMRP were indistinguishable between ΔGlued and wild-type extracts (Figure S3A). This observation provides further evidence that the role of BicD in regulating FMRP protein levels is not due to a general effect of inhibiting transport.

BicD is complexed with dynein and the plus end motor kinesin-1 on at least some bidirectional cargoes [19, 20], and a kinesin-1 family member associates with FMRP complexes and contributes to their transport in mammalian neurons [2]. In *Drosophila* primary neurons with a strong kinesin-1 heavy chain mutant genotype (*Khc^17/27^*), there was a striking alteration of FMRP appearance compared to wild-type cells, with discrete particles not detectable above the diffuse cytoplasmic signal (Movie S2B). This observation, which is reminiscent of the reduced size of a kinesin-1 mRNP cargo in *Drosophila* oocytes [21], raises the possibility that both dynein and kinesin-1 cooperate in FMRP transport in *Drosophila* neurons.

BicD may have a direct role as a constituent of FMRP:motor complexes. Alternatively, reduced levels of FMRP in *BicD* mutants might have an indirect effect by reducing the probability of FMRP encountering other transport factors. To attempt to discriminate between these possibilities, we took advantage of our observation that overexpression of BicD, even to a very large extent, does not alter the total amount of FMRP (Figure 2C). This presumably reflects wild-type levels of BicD being nonlimiting for the function in controlling FMRP levels.

2-fold overexpression of BicD (tagged with mCherry) dramatically increased the run lengths and net displacements of motile FMRP::GFP particles in cultured neurons, compared to the wild-type (Figures 3C and 3D). Run lengths in processes were similar for movements both toward and away from the cell body upon BicD overexpression (Figure S4D). Nonetheless, there was increased targeting of FMRP into distal processes compared to wild-type (Figures 3A and 3B). Once again, this presumably reflects the ability of long-distance, unbiased bidirectional transport to aid cargo spreading [17]. These results demonstrate that BicD is able to control motility and subcellular localization of FMRP independently from the role in regulating overall levels of the protein.

The results of quantification of particle motility, together with our observations that (1) FMRP is recruited by means of the domain of BicD involved in linking cargoes to dynein (Figures 1A and 1B) and (2) FMRP colocalizes in motile particles with BicD in vivo (Figure 1H; Movie S1), provides strong evidence that BicD plays a direct role in FMRP:motor complexes. In the case of other cargoes studied, BicD is not obligatory for their linkage to motor complexes but increases their travel distances significantly [8, 19, 22]. The residual directed transport of FMRP particles in *BicD* mutant neurons suggests that BicD may play a similar stimulatory role in this context. Other components of FMRP-containing transport particles presumably also contribute to linkage with motor proteins.

### BicD Cooperates with FMRP during Dendritic Morphogenesis

We next explored the functional significance of the BicD:FMRP interaction by focusing on the role of FMRP in dendritic morphogenesis [1]. We studied the well-characterized model system for dendritic development in the *Drosophila* third instar larva, the dorsal class IV dendritic arborization (da) neuron ddaC [23, 24].

**Figure 4:**
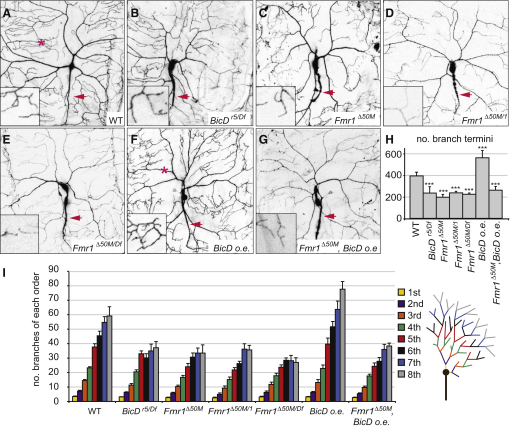
BicD Is Required for Correct Dendritic Morphogenesis and Requires FMRP to Induce Dendritic Branching (A–G) Representative confocal projections of dorsal ddaC neurons within segment A2 of third instar larvae, visualized with the class IV da-specific driver ppk-GAL4 and UAS-CD8::GFP. Red arrows show axons; other processes are dendritic. *BicD o.e.*, *UAS*-*BicD* overexpressed specifically in class IV da neurons with ppk-GAL4; *Df*, *Df(3R)BSC526*. Insets show higher magnification views of the typical density of dendritic termini. (H) Quantification of number of branch termini. (I) Quantification of the number of branches of each order (defined as in the schematic cut-away of a neuron [right]). In (H) and (I), the values for *Fmr1^Δ50M^* and *Fmr1^Δ50M^, BicD o.e.* are not significantly different (t test). Note that the terminal processes of neurons overexpressing BicD are frequently shorter than wild-type (e.g., compare regions near asterisks in A and F; terminal branches frequently extend back to this region in WT, but not in *BicD o.e.*). n = 6 or 7 neurons (from 4–7 larvae) for each genotype in (H) and (I). Error bars in (H) and (I) represent SEM; ^∗∗∗^p < 0.001 (t tests, compared to WT).

Dorsal ddaC neurons within zygotic *BicD* mutant larvae had a much less extensively branched dendritic arbor than wild-type cells (Figures 4A, 4B, 4H, and 4I). A similar inhibition of the dendritic branching program was observed in three different zygotic *Fmr1* null mutant genotypes (Figures 4C–4E, 4H, and 4I; *Fmr1^Δ50M^* homozygotes, *Fmr1^Δ50M/1^*, and *Fmr1^Δ50M^*^/*Df(3R)BSC526*^; see Supplemental Experimental Procedures for details of alleles). Intermediate terminal branching defects were also found in ddaC neurons heterozygous for *Fmr1^Δ50M^* (Figures S2D, S2F, and S2G). This phenotype, which could be suppressed by the FMRP::GFP transgene (Figures S2E and S2F), underscores the importance of correct FMRP protein levels for neuronal morphogenesis.

Our results demonstrate that both BicD and FMRP are required for efficient branching of the dendritic arbor in dorsal ddaC neurons. Interestingly, FMRP negatively regulates dendritic elaboration in mushroom body neurons in adult brains [25]. It has also previously been reported that mutating *Fmr1* increases branching of ventral da neurons [12], although effects on specific classes of neurons within the cluster were not reported. The differential requirements for *Fmr1* in controlling the morphology of different neuronal cells is consistent with previous findings. Morales et al. [26] showed that *Fmr1* mutations cause overextended axons in LNv cells but a failure of axon extension in DC neurons. Cell type-specific effects of FMRP on neuronal morphogenesis may reflect differences in the repertoire of its mRNA targets.

BicD overexpression specifically in class IV da neurons significantly increased the number of dendritic branches in the distal regions of arbors in dorsal ddaC neurons compared to wild-type (Figures 4F, 4H, and 4I). This result, together with the diminished branching in *BicD* mutant neurons, reveals a correlation between the amount of available BicD and the degree of arborization of ddaC and that BicD can function autonomously within neurons to control this process.

Strikingly, the ability of overexpressed BicD to augment dendritic branching of ddaC appears to be due predominantly to its interaction with FMRP, as evidenced by a strong suppression of the BicD overexpression phenotype in *Fmr1* null mutants, with neuronal morphology not significantly different to the *Fmr1* mutant alone (Figures 4G–4I). Because BicD overexpression does not alter FMRP protein levels, increased branching is likely to be influenced by BicD's ability to control FMRP motility. Our live cell imaging revealed that BicD promotes long-distance bidirectional transport of FMRP complexes on microtubules, thereby facilitating the exploration of neuronal processes. Such a mechanism may increase the probability of encounters of these complexes with factors that activate translation of associated mRNAs, which in some contexts could be responsive to local signaling [27]. Nonetheless, the reduction of overall FMRP protein levels is highly likely to contribute to *BicD* loss-of-function phenotypes in da neurons, as potentially is the altered transport of FMRP-independent cargoes.

## Supplemental Information

Document S1. Supplemental Experimental Procedures, Four Figures, and One Table

Movie S1. Cotransport of BicD::mCherry and FMRP::GFP in Cultured Primary Drosophila Neurons Consisting of two concatenated movies, A and B. (A) Primary cultured neuron from third instar larvae expressing UAS-FMRP::GFP (green; under C155-GAL4 control) and tub-BicD::Cherry (red). Extensive colocalization of BicD and FMRP in moving particles can be observed. Fluorescence outside the cell comes from debris in the culture medium. Movie consists of four loops; each loop represents 4 min 40 s. (B) Close-up of one particle (arrow), showing correspondence of the BicD and FMRP signals during movement in both directions. Movie consists of four loops; each loop represents 3 min 54 s.

Movie S2. Behavior of FMRP:GFP in Wild-Type, BicD, and Khc Mutant Cultured Primary Drosophila Neurons Consisting of two concatenated movies, A and B. (A) (Left) Primary cultured neuron from a third instar larva expressing UAS-FMRP::GFP (under C155-GAL4 control) in the wild-type background. Some FMRP particles (e.g., arrow) can be seen moving bidirectionally over long distances. (Right) Primary cultured neuron from a third instar larva expressing UAS-FMRP::GFP (under C155-GAL4 control) in the *BicD* mutant background. Some FMRP particles (arrows) are motile but they mostly perform relatively short, oscillatory movements. In this and other movies *BicD* mutant genotype is *r5/Df119*. Movies consist of four loops; each loop represents 2 min 10 s (left) and 2 min 8 s (right). Width of region shown is 14.7 μm and 24.6 μm for wild-type and *BicD* mutant, respectively. There are higher levels of FMRP::GFP signal in the cell body of the wild-type neuron (top left of image) than in the cell body of the *BicD* mutant neuron (flanked by arrows) at the onset of filming (i.e., before photobleaching). This reflects reduced amounts of total protein in nervous tissue (Figure 2B). (B) Primary cultured neuron from a third instar larva expressing UAS-FMRP::GFP (under C155-GAL4 control) in a kinesin-1 heavy chain strong hypomorphic background (*Khc^17^*^/*27*^). Discrete FMRP particles cannot be readily detected above the diffuse cytoplasmic signal. Movie consists of four loops; each loop represents 1 min 15 s. Width of region shown is 18 μm.

Movie S3. Behavior of FMRP:GFP in Wild-Type and BicD Mutant Chordotonal Organ Neurons In Situ Chordotonal organ neuronal cluster in filleted third instar larvae expressing UAS-FMRP::GFP (under C155-GAL4 control) in the wild-type and *BicD* mutant background. Dendrites and axons are above and below the cell body, respectively. Although most of the particles are stationary, it is possible to observe directed movement of some particles (arrows), which are less extensive in *BicD* mutants. See Figures 3E and 3F for quantification from multiple cells. The low frequency of movement of FMRP puncta even in wild-type cells is consistent with observations of neuronal mRNPs in other studies [7–9]. Movies consist of four loops; each loop represents 2 min 55 s (left) and 2 min 15 s (right). Width of region shown is 51.3 μm and 36.6 μm for WT and *BicD* mutant, respectively. N.B. reduced levels of FMRP::GFP signal overall in *BicD* mutant neurons, reflecting reduced amounts of total protein in nervous tissue. These movies are of projections of four z-sections of ∼1 μm each. Individual z-slices were used for the quantitative data in Figures 3E and 3F, permitting more rapid imaging of particles. Fluorescence outside of neurons in the wild-type results from autofluorescence in oenocytes. The oenocytes are visible in a subset of projections of both wild-type and mutant preparations, because of variability in fillet dissection and mounting.
