# Supplementary material for: Bicaudal-D Regulates Fragile X Mental Retardation Protein Levels, Motility, and Function during Neuronal Morphogenesis
Source: Curr Biol. 2010 Aug 24;20(16):1487–92. doi: 10.1016/j.cub.2010.07.016 (PMC2927779; doi:10.1016/j.cub.2010.07.016)
Supplement: Document S1. Supplemental Experimental Procedures, Four Figures, and One Table [file mmc1.pdf]

Current Biology, Volume *20*

Supplemental Information

Bicaudal-D Regulates Fragile X Mental Retardation Protein Levels, Motility, and Function during Neuronal Morphogenesis

Ambra Bianco, Martin Dienstbier, Hannah K. Salter, Graziana Gatto, and Simon L. Bullock

Inventory of supplemental information – Bianco et al.

Supplemental experimental procedures

Supplemental references

Figure S1 (related to figure 1)

Figure S2 (related to figures 1, 3 and (mostly) 4)

Figure S3 (related to figure 2)

Figure S4 (related to figure 3)

Table S1 (related to figure 1)

Movies S1-S3 titles and legends; movie files available online

Supplemental Experimental Procedures

Drosophila strains

Zygotic *BicD* null larvae are trans-heterozygous for a *BicD* null allele (*r5*) that introduces a translational stop after codon 50 of 782 [1] and a chromosomal deficiency that removes the entire *BicD* locus (*Df(2L)TW119*, also known as *Df119*). As reported previously [1, 2], the vast majority of null mutants fail to eclose, with those that do dying in the next few hours. Survival and fertility of the *trans*-heterozygotes is fully rescued by BicD transgenes [2, 3], indicating that the observed defects do not arise from additional mutations in the background. *UAS-Fmr1::GFP* flies where kindly provided by Fen-Biao Gao [4]. Even with strong GAL4 drivers we observed relatively weak overexpression of FMRP::GFP, constituting only ~20% of the amount of endogenous FMRP. The null allele *Fmr1*$^{\Delta 50M}$ [5] was obtained from the Bloomington *Drosophila* Stock Center. This is a P-element excision allele removing flanking DNA, including the 5' non-coding exons and the first coding exon, of the *Fmr1* gene. The null allele *Fmr1*^{1} was kindly provided by Fen-Biao Gao [4]. This allele has an 11-nucleotide deletion that, owing to the frame shift, results in a stop codon after amino acid 126. The chromosomal defiency *Df(3R)BSC526*, which uncovers the *Fmr1* locus, was obtained from the Bloomington *Drosophila* Stock Center.

Egl::GFP, driven from a ubiquitously-expressed tubulin promoter, and UAS-BicD are described in [3] and [6], respectively. The *tub-BicD::mCherry* construct was generated by modifying the *tub-BicD::eGFP* construct described in [3]. The BicD::mCherry fusion fully rescues survival and fertility of *BicD*$^{r5/Df119}$ animals. Gal4 drivers used were *elav*^{C155} (pan-neuronal; Bloomington *Drosophila* Stock Center) and *ppk1.9* (Class IV da neurons; kindly provided by Darren Williams [7]). *Dhc64C* alleles are described in [8]; *Khc* alleles are described in [9]. UAS-EB1::GFP was a kind gift of Hiro Okhura [10].

To assay BicD and FMRP colocalization and FMRP motility upon mild BicD overexpression, *elav*^{C155}*-GAL4* flies were crossed with *w*; *tubBicD::mCherry; UAS-Fmr1::GFP*. FMRP motility and protein levels in wild-type and *BicD* mutants were assessed using third instar larval brains from the following crosses: (1) *elav*^{C155}*-GAL4*

crossed to *UAS-Fmr1::GFP* and (2) *w, elav*^{C155}*-GAL4*; *BicD*^{Df119}/*CyO Actin:GFP* crossed to *w*; *BicD*^{r5}/*CyO Actin:GFP*; *UAS-Fmr1::GFP/TM6b,Tb*. To show that FMRP levels are not affected by even very strong *BicD* overexpression, *elav*^{C155}*-GAL4*; *UAS-BicD* was crossed to *UAS-Fmr1::GFP*.

The following crosses were used for the analysis of dendritic morphology in ddaC neurons in figure 4: (1) *BicD*^{Df119}/*CyO Actin:GFP; ppk1.9-GAL4, UAS-CD8::GFP* crossed to *w*; *BicD*^{r5}/*CyO Actin:GFP*, (2) *UAS-CD8::GFP; UAS-BicD; Fmr1*$^{\Delta 50M}$*/ TM6b,Tb* crossed to *ppk1.9-GAL4; Fmr1*$^{\Delta 50M}$*/ TM6b,Tb*, (3) *UAS-CD8::GFP;; Fmr1*$^{\Delta 50M}$*/ TM6b,Tb* crossed to *ppk1.9-GAL4; Fmr1*$^{\Delta 50M}$*/ TM6b,Tb*, (4) *ppk1.9-GAL4; Fmr1*$^{\Delta 50M}$*/ TM6b,Tb* crossed to *UAS-CD8::GFP; Df(3R)BSC526/TM6b,Tb*, (5) *ppk1.9-GAL4; Fmr1*$^{\Delta 50M}$*/ TM6b,Tb* crossed to *UAS-CD8::GFP; Fmr1*^{1}*/TM6b,Tb.*

Primary neuronal cell culture

Third instar larval brains were isolated, disaggregated and cells cultured as previously described in [11]. Cells appear round immediately after plating and differentiate during the following three to four days. Consistent with previous observations [11], we were unable to observe a clearly differentiated axon in the vast majority of cells. Cells were analyzed after 5 to 7 days of culture.

***In vivo* imaging**

Third instar larvae were dissected and mounted in Ca^{2+}-free saline (130 mM NaCl, 36 mM sucrose, 5 mM KCl, 4 mM $MgCl_2$, 2 mM $CaCl_2$, 5 mM HEPES (pH 7.3) and 0.5 mM EGTA) to prevent muscle contraction. Primary neurons were plated in Lab-Tek chambered cover glasses (Thermo Scientific). Larvae and cells were imaged at 22-23°C using a spinning disk UltraVIEW ERS confocal microscope (PerkinElmer) using a 63/1.4NA oil-immersion PlanApo Olympus objective and an Orca ER CCD camera (Hamamatsu). Image series, from a single focal plane, lasted from 2 to 6 min both for cells and larvae. Larval fillets were imaged only during the first 20 min following dissection, although robust cargo transport in these and other neurons was maintained for at least 30 min after dissection. Chordotonal organ neurons were imaged because, unlike

in other larval neuronal cell types in situ, it was possible to image and analyze FMRP::GFP transport due to their relatively simple shape, which permits filming in a single focal plane.

Analysis of FMRP::GFP motility

Motile FMRP::GFP particles were tracked manually using ImageJ and run length, net movement and velocity calculated using Microsoft Excel. Run length was defined as the distance a particle moves before a reversal or a pause. Net displacement was defined as the total direct distance a particle moves during the whole time of observation.

Mean run length was calculated by averaging the values for all runs for a given genotype (i.e. considering the different movements of the same particle as independent events). The mean velocity of ~ 0.2 μm/s, stated in the legend to figure 1, was also calculated by averaging the values for all runs for a given genotype. Mean net displacement was calculated by averaging the net displacement of each individual particle. The data in figure 3 quantifying FMRP::GFP motility in neuronal culture were obtained from 40 to 60 movies per genotype. 6 to 10 independent primary cultures were prepared per genotype. Quantification of motility in chordotonal organs was based on imaging 12 and 18 *BicD* mutant and wild-type larval fillets, respectively.

GST pull downs, immunoprecipitation from embryo extracts and yeast two hybrid assays

The K730M substitution is encoded by the $BicD^{r11}$ allele [1]. In heteroallelic combinations with *BicD* null mutant alleles, $BicD^{r11}$ behaves as a null allele based on phenotypic analysis [1]. As described above, only a very small proportion of *BicD* null mutants eclose from the pupal case, with those that do dying in the next few hours [1, 2]. Thus, K730M mutant material cannot be readily used for biochemistry. We therefore previously assayed the effect of this substitution on Egl and Rab6 binding by expressing epitope-tagged versions of wild-type or K730M BicD in egg chambers in the presence of endogenous BicD and/or with binding assays with recombinant proteins or the yeast two-hybrid system [3].

GST pull downs were conducted as described [3]. Briefly, 3 μg of GST-fused wild-type and K730M BicD CTD (aa 538-782) or GST protein alone was coupled to 5 μl of glutathione sepharose beads. These beads were incubated with 600 μl of 0 – 18 h embryonic extract (prepared in DXB buffer as described [3] and pre-cleared with gluthathione sepharose) by rotating for 1 h at 4°C. Following 2 washes in DXB buffer and 3 washes in PBS, samples were resuspended in 50 μl LDS sample buffer, electrophoresed on a 4-12% Bis-Tris gel (Invitrogen) and analyzed by Coomassie staining or western blotting. Immunoprecipitation with anti-BicD ([12]; obtained from the Developmental Studies Hybridoma Bank (DSHB) developed under the auspices of the NICHD and maintained by The University of Iowa, Department of Biology, Iowa City, IA 52242) was as described [3]. RNase treatments used 5 μl of RNase cocktail (RNaseA and RNase T1 (Ambion)) per 100 μl of extract. 5 μl of RNase inhibitor (Promega) was added per 100 μl of extract in controls for these experiments. RNase positive and negative samples were incubated at 30°C for 20 min. In control experiments, RNA was purified from extracts following such treatments; RNA was fully degraded by the RNase treatment, whereas RNA integrity was not affected in the RNase negative extract.

Yeast two-hybrid assays used the vectors pGAD424 and pGBDU-C1 [13]. pGAD424 carries the LEU2 marker gene, and pGBDU-C1 carries the URA3 marker gene. Fragments of *Drosophila* BicD or FMRP were PCR-amplified and cloned in frame with the Gal4p transactivation domain in pGAD424 or the Gal4p DNA binding domain in pGBDU-C1, respectively. The integrity of all inserts was confirmed by sequencing. Plasmids were transformed into the yeast two-hybrid strains PJ69-4a or PJ69-4α [13], and pairs of BicD and FMRP fragments introduced into the same cells by mating. Successful mating was confirmed by plating on –LEU/–URA medium. Interactions between the bait and prey, which result in expression of HIS3 and ADE2 were assayed by plating on –HIS/–LEU/–URA medium for 72-96 hours. Due to the relatively weak interaction of FMRP and the BicD CTD, growth conditions were made less stringent by including adenine in the medium.

Liquid chromatography mass spectrometry/mass spectrometry

Gel bands were excised, washed, alkylated, and in-gel digested with trypsin. The extracted peptides were separated by nanoscale liquid chromatography (LC Packings, Armsterdam, Netherlands) on a reverse phase C18 column (150 X 0.075 mm ID, flow rate 0.25 μl/min). The eluate was introduced directly into a Q-STAR hybrid tandem mass spectrometer (MDS Sciex, Ontario, Canada). Spectra were searched against a NCBI non-redundant database with MASCOT MS/MS Ions search (www.matrixscience.com).

Western blotting of larval brain extracts

Larval brains were dissected and crushed with a pestle on ice in a 1.5 ml tube. Extracts were then denatured via boiling in LDS and separated on NuPAGE 4-12% Bis-tris precast gels (Invitrogen) or transferred onto PVDF membranes for immunoblotting. For western blotting, the following primary antibodies were used: mouse anti-BicD 1B11 (1:500); mouse anti-FMRP 5A11 and 5B6 (1:500) (all from DHSB); rabbit anti-β-actin (1:3000; Abcam) and rabbit anti-Egl (1:5000; [14] (a kind gift of Caryn Navarro and Ruth Lehmann). HRP-conjugated secondary antibodies and ECL reagent (Amersham) were used for detection.

Immunostaining and detection of mitochondria

Dissection of third instar larval neuromuscular system fillets was performed as previously described [15]. Larvae were dissected in Ca^{2+}-free solution and fillets fixed immediately afterwards (15 min in 4% paraformaldehyde in PBS). Samples were then blocked for 1 h at room temperature in 5% bovine serum albumin (Sigma) in PBS/0.1% NP-40. Primary antibodies (mouse anti-GFP (1:500; Roche) and rabbit anti-HRP (1:300; Jackson Immunoresearch)) were applied overnight at 4°C and secondary, fluorescently-conjugated antibodies were incubated for 1 h at room temperature. 6 washes of 15 min each with PBS/0.1% NP-40 were conducted after antibody incubations.

Dorsal ddaC neurons were imaged with a Biorad Radiance or Zeiss 710 confocal microscope using a 25x/0.8 NA oil immersion or a 20x/0.8NA air objective. Images shown in figures 4 and S2 are a projection of 30 to 40 z-sections. ddaC neurons are an established model to study neuronal morphology as they present the following

advantages: 1) they are class IV neurons, i.e. they harbour the highest level of morphological complexity that can be found in the larval epithelium, thus allowing the scoring of detailed aspects of neuronal shape and 2) ddaC neurons are the only class IV neurons present in the dorsal side of a larva; it is therefore possible to correctly attribute dendritic arbors to the cell body they originated from, allowing a single neuron to be scored.

For immunofluorescence of primary neurons, cells were fixed in 4% formaldehyde for 10 min. BicD was detected using antibodies raised in rabbit against amino acids 1-543 of *Drosophila* BicD, expressed in bacteria and purified using an N-terminal His tag. FMRP was detected using a mixture of mouse anti-FMRP 5A11 and 5B6 (1:500). Samples were imaged using a Zeiss 710 confocal microscope.

For analysis of the distribution of mitochondria in primary neurons, cells were cultivated for 5 days after plating and then incubated with 4 nM Mitotracker Green-FM (Invitrogen) for 15 min, washed twice with fresh medium and imaged.

Quantitative RT-PCR (RT-qPCR)

cDNA was synthesized from total RNA (2.5 μg) from third instar larval brains using random hexamer primers and Superscript III (Invitrogen). RT-qPCR reactions were carried out with the ABI7900 Taqman thermocycler (Applied Biosystems). Reaction mixtures for amplification contained 0.5 μl of each gene expression assay (AB Biotech), 5 μl of 2x Master Mix (AB Biotech), 1 μl of cDNA (~50 ng) and water to 10 μl.

The gene expression assays used were *Fmr1* (Dm02136378) and *β-actin* (Dm02361909) (http://www3.appliedbiosystems.com/AB_Home/index.htm). Values for gene expression in each case were calculated relative to a standard curve of *Fmr1* expression whose consistency from different samples was confirmed in each case using *β-actin* expression. For each genotype, duplicate experiments were performed for each of 3 different RNA preparations.

RNAi experiments in cultured D-mel cells

Portions of the coding regions of BicD and GFP were amplified with flanking T7 promoter sequences. Predicted off-target free amplicons (*Drosophila* RNAi Screening

Center; http://www.flyrnai.org) were generated using the following primer pairs (T7 sequences are lower case):

BicD:

T7BicDRNAiF: taatacgactcactatagggCGAACTTAAATCTCCTGACGGCAC

T7BicDRNAiR: taatacgactcactatagggAGATGTGGGTAAGAGTTTGAACATC

GFP:

T7GFPRNAiF: taatacgactcactatagggCTTCAGCCGCTACCCCGACCAC

T7GFPRNAiR: taatacgactcactatagggCAGCACGGGGCCGTCGC

The amplicons were used to generate dsRNAs using standard protocols. For each treatment, 2 x 10^6 D-mel cells (Invitrogen; a derivative of S2 cells that grows in serum-free medium) were treated with 30 μg of dsRNA for 4 days. Cells were then lysed, and proteins analyzed by western blotting with chemiluminescent detection.

Supplemental References

1. Ran, B., Bopp, R., and Suter, B. (1994). Null alleles reveal novel requirements for Bic-D during *Drosophila* oogenesis and zygotic development. Development *120*, 1233-1242.
2. Li, X., Kuromi, H., Briggs, L., Green, D.B., Rocha, J.J., Sweeney, S.T., and Bullock, S.L. (2010). Bicaudal-D binds clathrin heavy chain to promote its transport and augments synaptic vesicle recycling. EMBO J *29*, 992-1006.
3. Dienstbier, M., Boehl, F., Li, X., and Bullock, S.L. (2009). Egalitarian is a selective RNA-binding protein linking mRNA localization signals to the dynein motor. Genes Dev *23*, 1546-1558.
4. Lee, A., Li, W., Xu, K., Bogert, B.A., Su, K., and Gao, F.B. (2003). Control of dendritic development by the *Drosophila* fragile X-related gene involves the small GTPase Rac1. Development *130*, 5543-5552.
5. Zhang, Y.Q., Bailey, A.M., Matthies, H.J., Renden, R.B., Smith, M.A., Speese, S.D., Rubin, G.M., and Broadie, K. (2001). *Drosophila* fragile X-related gene regulates the MAP1B homolog Futsch to control synaptic structure and function. Cell *107*, 591-603.
6. Bullock, S.L., Nicol, A., Gross, S.P., and Zicha, D. (2006). Guidance of bidirectional motor complexes by mRNA cargoes through control of dynein number and activity. Curr Biol *16*, 1447-1452.
7. Ainsley, J.A., Pettus, J.M., Bosenko, D., Gerstein, C.E., Zinkevich, N., Anderson, M.G., Adams, C.M., Welsh, M.J., and Johnson, W.A. (2003). Enhanced

locomotion caused by loss of the *Drosophila* DEG/ENaC protein Pickpocket1. Curr Biol *13*, 1557-1563.

8. Gepner, J., Li, M., Ludmann, S., Kortas, C., Boylan, K., Iyadurai, S.J., McGrail, M., and Hays, T.S. (1996). Cytoplasmic dynein function is essential in *Drosophila* melanogaster. Genetics *142*, 865-878.
9. Brendza, K.M., Rose, D.J., Gilbert, S.P., and Saxton, W.M. (1999). Lethal kinesin mutations reveal amino acids important for ATPase activation and structural coupling. J Biol Chem *274*, 31506-31514.
10. Satoh, D., Sato, D., Tsuyama, T., Saito, M., Ohkura, H., Rolls, M.M., Ishikawa, F., and Uemura, T. (2008). Spatial control of branching within dendritic arbors by dynein-dependent transport of Rab5-endosomes. Nat Cell Biol *10*, 1164-1171.
11. Estes, P.S., O'Shea, M., Clasen, S., and Zarnescu, D.C. (2008). Fragile X protein controls the efficacy of mRNA transport in *Drosophila* neurons. Mol Cell Neurosci *39*, 170-179.
12. Suter, B., and Steward, R. (1991). Requirement for phosphorylation and localization of the Bicaudal-D protein in *Drosophila* oocyte differentiation. Cell *67*, 917-926.
13. James, P. (2001). Yeast two-hybrid vectors and strains. Methods Mol Biol *177*, 41-84.
14. Mach, J.M., and Lehmann, R. (1997). An Egalitarian-BicaudalD complex is essential for oocyte specification and axis determination in *Drosophila*. Genes Dev *11*, 423-435.
15. Kuromi, H., and Kidokoro, Y. (1998). Two distinct pools of synaptic vesicles in single presynaptic boutons in a temperature-sensitive *Drosophila* mutant, shibire. Neuron *20*, 917-925.
16. Bullock, S.L., and Ish-Horowicz, D. (2001). Conserved signals and machinery for RNA transport in *Drosophila* oogenesis and embryogenesis. Nature *414*, 611-616.
17. Hoogenraad, C.C., Wulf, P., Schiefermeier, N., Stepanova, T., Galjart, N., Small, J.V., Grosveld, F., de Zeeuw, C.I., and Akhmanova, A. (2003). Bicaudal D induces selective dynein-mediated microtubule minus end-directed transport. EMBO J *22*, 6004-6015.
18. Morales, J., Hiesinger, P.R., Schroeder, A.J., Kume, K., Verstreken, P., Jackson, F.R., Nelson, D.L., and Hassan, B.A. (2002). *Drosophila* fragile X protein, DFXR, regulates neuronal morphology and function in the brain. Neuron *34*, 961-972.
19. Knowles, R.B., and Kosik, K.S. (1997). Neurotrophin-3 signals redistribute RNA in neurons. Proc Natl Acad Sci U S A *94*, 14804-14808.
20. Kohrmann, M., Luo, M., Kaether, C., DesGroseillers, L., Dotti, C.G., and Kiebler, M.A. (1999). Microtubule-dependent recruitment of Staufen-green fluorescent protein into large RNA-containing granules and subsequent dendritic transport in living hippocampal neurons. Mol Biol Cell *10*, 2945-2953.

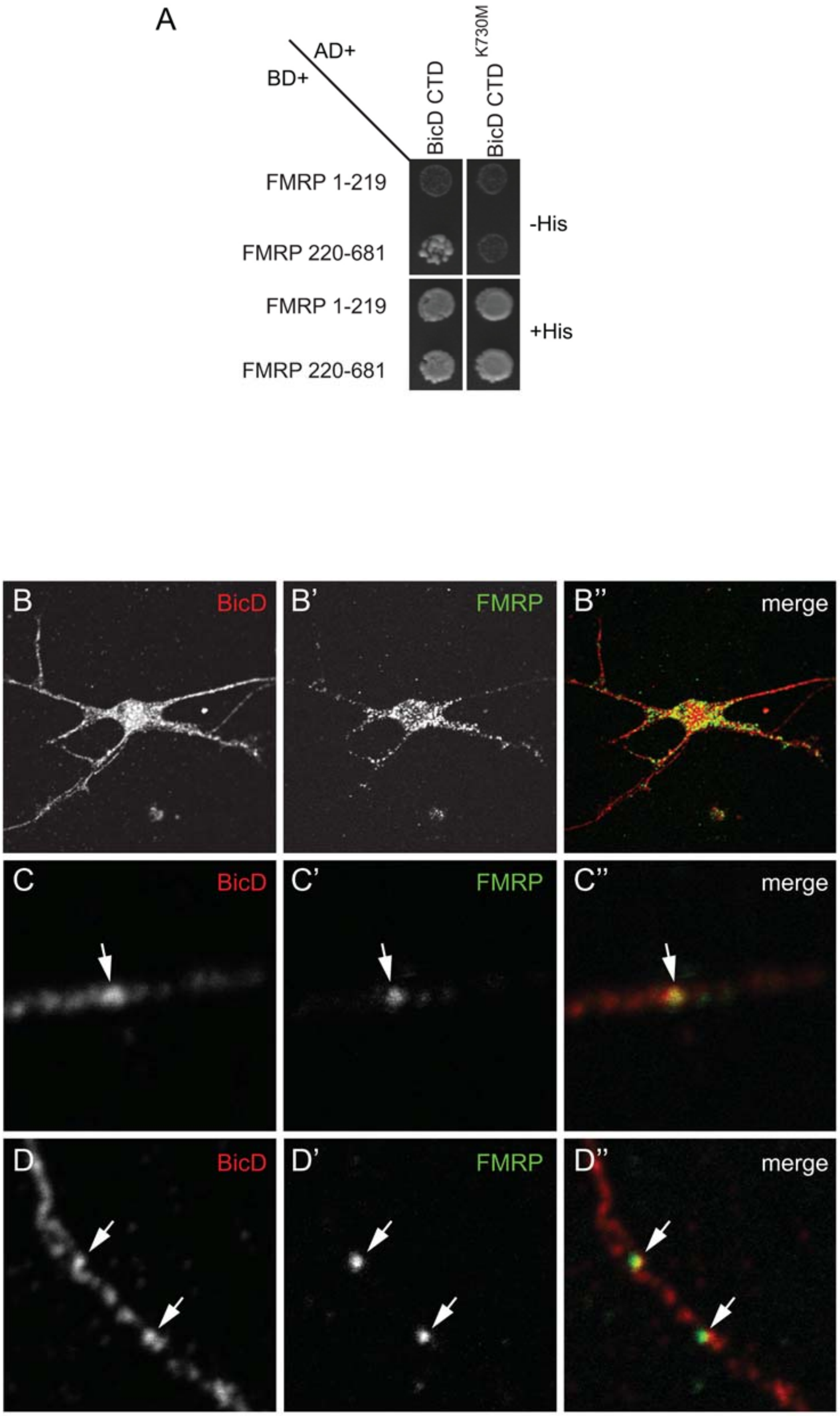

Figure S1. Probing potential BicD-FMRP interactions

(A) BicD CTD interacts specifically with an FMRP fragment in the yeast two-hybrid assay. Yeast two-hybrid assay showing interaction between the BicD CTD and the C-

terminal two-thirds of FMRP that is abolished by the K730M mutation in the BicD CTD. Strains containing interacting pairs of constructs reconstitute biosynthesis of His. Each strain exhibits equivalent growth in the presence of His. AD, GAL4 activation domain fusion; BD, GAL4 binding domain fusion. Note that, unlike when the Egl:BicD interaction is assayed [3], the interaction of FMRP and BicD is not strong enough to elicit growth under more stringent conditions (i.e. when adenine is also omitted from the medium).

(B) Immunostaining of endogenous BicD and FMRP in cultured larval neurons. BicD was detected with a Cy5-conjugated secondary antibody and FMRP with an Alexa488-conjugated secondary antibody. (A) Low magnification image of cell body and neurites. (B, C) Higher magnification views of neurites, showing examples of co-incidence of punctate FMRP signals and BicD signals (arrows). However, due to the very broad distribution of BicD in neurons it is not possible to draw a meaningful conclusion about the overall extent of complex formation between FMRP and BicD from these experiments (i.e. BicD signal would overlap with many other signals in fixed neurons just by chance; see main text for details of time-lapse imaging of co-transport of FMRP::GFP and BicD::mCherry). Note that BicD also has a much wider distribution than its cargoes on other cell types [16, 17] (MD and SLB, unpublished observations). This presumably reflects a sizeable pool of an auto-inhibited form of the protein: in the absence of binding of a cargo, the CTD engages in an interaction with an N-terminal region, thus preventing recruitment of the dynein/dynactin complex to the N-terminal region [17]. It is also possible that BicD transports other cargoes in these cells and that this contributes to the broad distribution of BicD relative to FMRP.

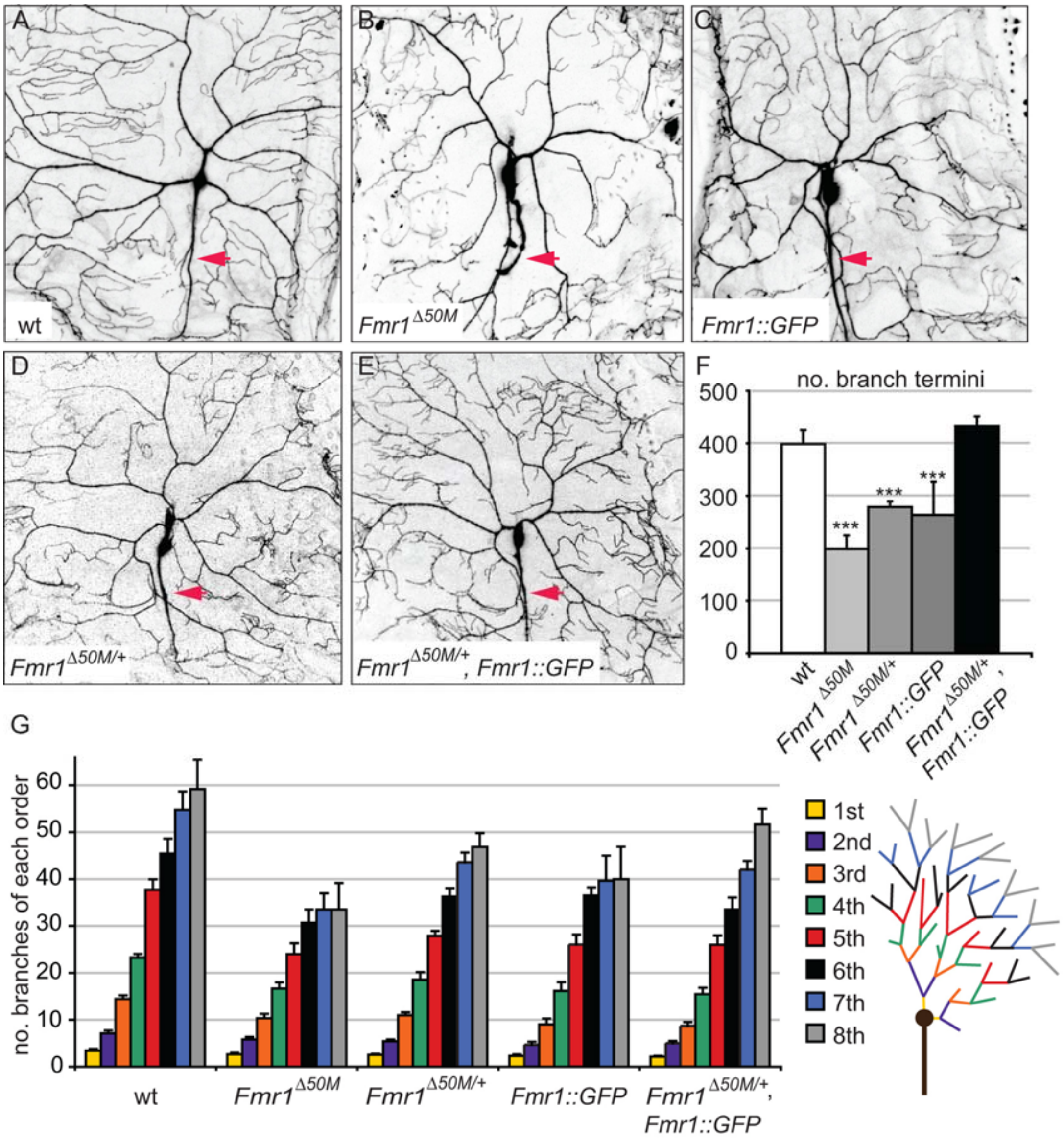

Figure S2. FMRP::GFP is functional *in vivo*

(A – E) Representative images of dorsal ddaC dendritic arborization (da) neurons in segment 2 of third instar larvae of the indicated genotypes (visualized with UAS-CD8::GFP expressed under the control of the class IV da neuron-specific driver ppk-GAL4). Red arrows indicate axon; other processes are dendritic.

(F) Quantification of terminal branch number in the dendritic arbor. Number of terminal branches is reduced in *Fmr1*$^{\Delta 50M}$ homozygous mutant and heterozygous larvae. There are significantly more branches in heterozygous than in homozygous mutant larvae ($P < 0.0004$ (*t-test*)). Branching is also reduced, compared to wild-type, following overexpression of *Fmr1::GFP* under control of ppk-GAL4 in the presence of wild-type levels of endogenous FMRP (i.e. *Fmr1*$^{+/+}$) (***, $P < 0.001$; *t-test*). Similar effects on neuronal morphology of *Fmr1* loss-of-function and overexpression have previously been reported in other neurons; Morales et al. [18] found that both *Fmr1* null mutations and

overexpression caused reduced extension of the axons of DC neurons in the adult *Drosophila* brain. The authors concluded that dose of FMRP may be under stringent regulation and that precise levels are required for proper function. Our results in dorsal ddaC neurons support this conclusion. ppk-GAL4 driven expression of *Fmr1::GFP* fully rescues terminal branch number defects in *Fmr1*$^{+/-}$ ddaC neurons. Thus, the FMR1::GFP fusion protein is functional. Note that is has previously been reported that terminal branch number in larval da neurons is increased in *Fmr1* mutants [4]. However, these authors analyzed the entire cluster of da neurons on the ventral side, whereas we analyzed the phenotype in a single neuron (ddaC; the only class IV da neuron in the dorsal cluster). The differential requirements for *Fmr1* in controlling the morphology of different neuronal cells is consistent with previous findings in *Drosophila* brains. Morales et al. [18] showed that *Fmr1* mutations cause overextended axons in LNv cells, but a failure of axon extension in DC neurons. Cell type-specific effects of FMRP on neuronal morphogenesis may reflect differences in the repertoire of its mRNA targets.

(G) Quantification of branch order of ddaC neurons. Number of branches of each order is reduced in *Fmr1*$^{\Delta 50M}$ null mutant and heterozygous larvae, and also following overexpression of *Fmr1::GFP* under control of ppk-GAL4 in the presence of wild-type levels of endogenous FMR1 (i.e. *Fmr1*$^{+/+}$). Overexpression of *Fmr1::GFP* in ddaC does not significantly suppress branch order defects in *Fmr1* heterozygotes or the reduced length of terminal branches (compare (E) with (A)); this could presumably reflect sub-optimal levels of *Fmr1* transgene expression. Indeed, given the stringent requirements for FMRP levels revealed by our previous experiments, we would have been surprised if all branching defects in *Fmr1*$^{-/+}$ neurons could be reverted to the wild-type by our *Fmr1* overexpression. In F and G, 6 to 7 neurons (from 4 – 7 larvae) were analyzed for each genotype. Error bars represent s.e.m..

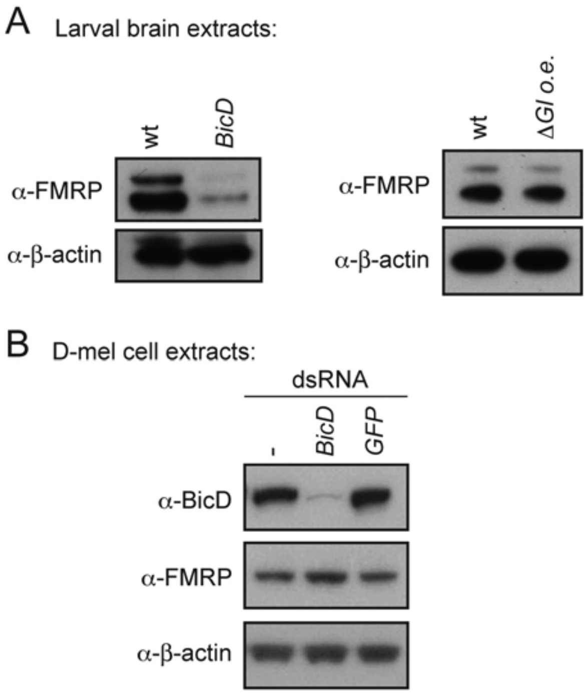

Figure S3. Endogenous FMRP levels in *BicD* mutant and ΔGl-expressing larval brains and following depletion of BicD in D-mel tissue culture cells by RNAi.

(A) Western blot on third instar larval brain extracts of the indicated genotypes, immunoblotted with anti-FMRP and anti-β-actin as a loading control. Endogenous levels of FMRP are decreased in *BicD* mutant brains (*BicD*$^{r5/Df119}$) even in the absence of expression of FMRP::GFP (compare with figure 2B; see legend to figure 2B for quantification). Endogenous levels of FMRP are not effected by overexpression of ΔGl pan-neuronally (using C155-GAL4).

(B) Depletion of BicD by RNAi in cultured D-mel cells does not reduce levels of endogenous FMRP. In control experiments, cells were either not treated with dsRNA (-) or treated with a dsRNA derived from GFP. For each treatment, 2 x 10^6 cells were incubated with 30 μg of dsRNA for 4 days. β-actin acts as a loading control. The blot shown is representative of two independent experiments. Note that the lower abundance FMRP isoform present in larval brain extracts was not detectable in these D-mel cell extracts. In larval brain extracts, the abundance of both isoforms is dependent on BicD (A).

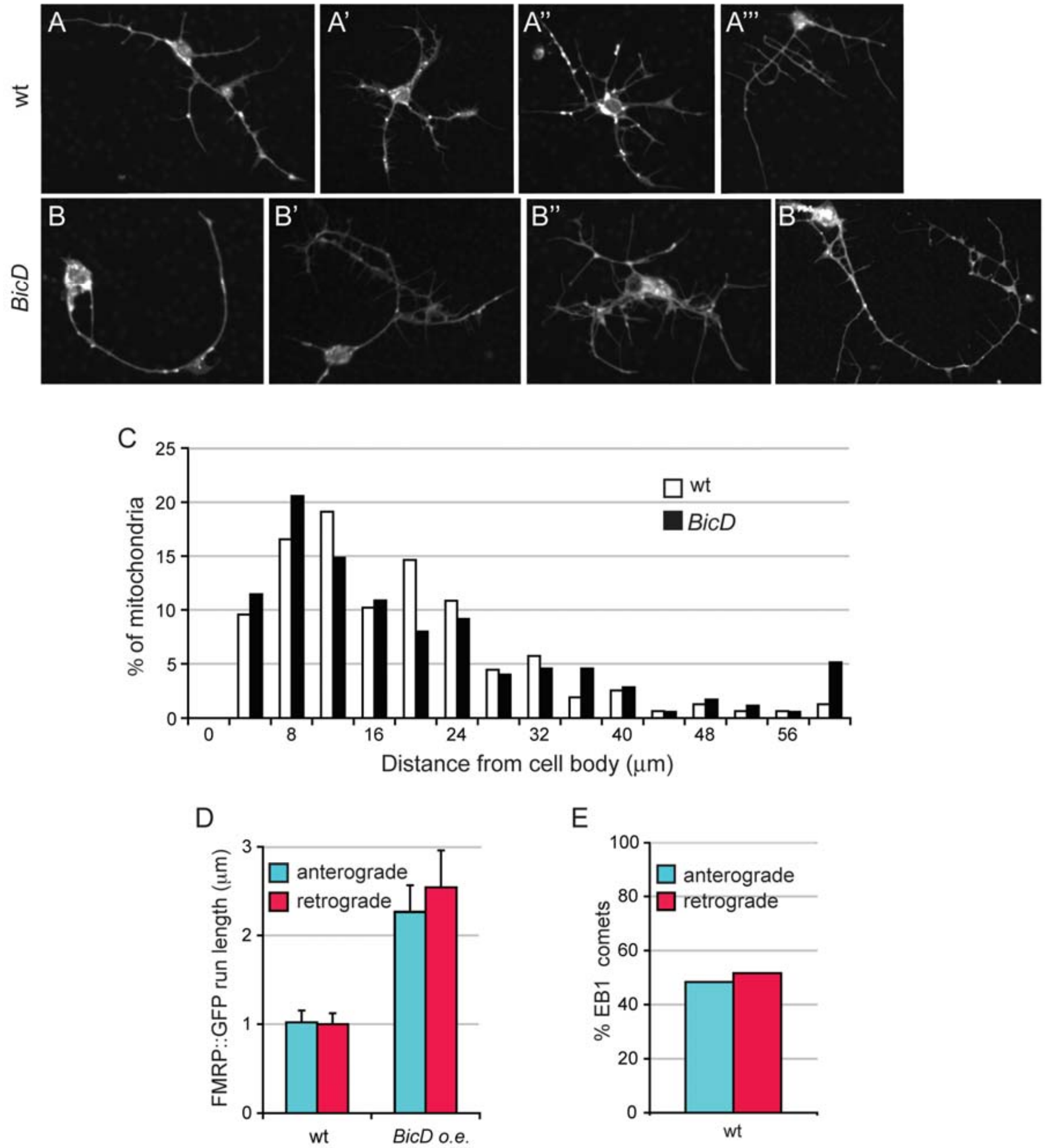

Figure S4. Characterization of *BicD* mutant primary neuronal cultures.
Examples of mitotracker-stained wt (A) and *BicD* mutant (*BicD*$^{r5/Df119}$) (B) third instar larval neurons in primary culture for 5 days. Overall there was no overt difference in morphology between these two genotypes; cellular morphology was as variable between individual cells of the same genotype as it was between individual cells of the different genotypes. (C) Quantification of mitochondrial distribution in neurites of neuronal primary cultures, reveals no significant difference between wild-type and *BicD* mutants. n = 157 mitochondria in 25 cells for wild-type and 175 mitochondria in 28 cells for *BicD*

mutant. Mitochondria are transported bidirectionally along microtubules in the *Drosophila* embryo independently of BicD (SLB, unpublished observations). (D) Quantification of anterograde and retrograde run lengths of FMRP::GFP in wild-type (wt) and BicD overexpressing (o.e.) primary cultured neurons. Errors bars are s.e.m.. (E) Quantification of microtubule polarity in wild-type primary cultured neurons, assayed by filming the plus-end-tracking marker EB1::GFP (under C155-GAL4 control) [10]. A similar number of microtubules grow away (anterograde) and toward (retrograde) the cell body. n = 306 comets in total, from 27 cells.

Table S1. Yeast two hybrid interactions tested between BicD and FMRP

	pGAD424 empty vector	pGAD424-BicD 1-543aa	pGAD424-BicD 536-782aa	pGAD424-BicD K730M 536-782aa [(c)]
pGBDU-C1 empty vector	-	-	-	-
pGBDU-C1-FMRP 1-681aa (FL) [(a)]	-	-	-	-
pGBDU-C1-FMRP 1-219aa	-	-	-	-
pGBDU-C1-FMRP 220-618aa	-	-	+	-
pGBDU-C1-FMRP 220-417aa [(b)]	+/-	+/-	+/-	+/-
pGBDU-C1-FMRP 418-681aa	-	-	-	-

+, growth in the absence of histidine; -, no growth in the absence of histidine; pGAD424, activation domain vector; pGBDU-C1, binding domain vector; FL, full length protein. Scored 96 h after plating. Note that, unlike when the Egl:BicD interaction is assayed [3], the interaction of FMRP and BicD is not strong enough to elicit growth under more stringent conditions (i.e. when adenine is also omitted from the medium).

a, the inability of full length FMRP to interact with the BicD CTD could reflect these FMRP sequences not folding in the context of the fusion with the DNA binding domain or an autoinhibited conformation that is relieved in the 220 – 618 fragment by removing the N-terminal sequences. This truncated FMRP fragment could conceivably mimic a conformational change that occurs in *Drosophila* and contributes to forming a stable FMRP:BicD complex.

b, there was very weak growth (+/-) even with the empty activation domain vector. The extent of growth was not detectably augmented by the FMRP sequences, suggesting that there is no significant interaction between FMRP 220-417 and BicD 536-782aa.

c, the K730M mutation does not inhibit binding of the BicD CTD to other copies of BicD in the yeast two hybrid assay [3], indicating that is does not have a general toxic effect on protein folding or stability.

Movie S1. Cotransport of BicD::mCherry and FMRP::GFP in Cultured Primary *Drosophila* Neurons

Consisting of two concatenated movies, A and B.

(A) Primary cultured neuron from third instar larvae expressing UAS-FMRP::GFP (green; under C155-GAL4 control) and tub-BicD::Cherry (red). Extensive colocalization of BicD and FMRP in moving particles can be observed. Fluorescence outside the cell comes from debris in the culture medium. Movie consists of four loops; each loop represents 4 min 40 s.

(B) Close-up of one particle (arrow), showing correspondence of the BicD and FMRP signals during movement in both directions. Movie consists of four loops; each loop represents 3 min 54 s.

Movie S2. Behavior of FMRP:GFP in Wild-Type, *BicD*, and *Khc* Mutant Cultured Primary *Drosophila* Neurons

Consisting of two concatenated movies, A and B.

(A) (Left) Primary cultured neuron from a third instar larva expressing UAS-FMRP::GFP (under C155-GAL4 control) in the wild-type background. Some FMRP particles (e.g., arrow) can be seen moving bidirectionally over long distances. (Right) Primary cultured neuron from a third instar larva expressing UAS-FMRP::GFP (under C155-GAL4 control) in the *BicD* mutant background. Some FMRP particles (arrows) are motile but they mostly perform relatively short, oscillatory movements. In this and other movies *BicD* mutant genotype is *r5/Df119*. Movies consist of four loops; each loop represents 2 min 10 s (left) and 2 min 8 s (right). Width of region shown is 14.7 μm and 24.6 μm for wild-type and *BicD* mutant, respectively. There are higher levels of FMRP::GFP signal in the cell body of the wild-type neuron (top left of image) than in the cell body of the *BicD* mutant neuron (flanked by arrows) at the onset of filming (i.e., before photobleaching). This reflects reduced amounts of total protein in nervous tissue (main text Figure 2B).

(B) Primary cultured neuron from a third instar larva expressing UAS-FMRP::GFP (under C155-GAL4 control) in a kinesin-1 heavy chain strong hypomorphic background ($Khc^{17/27}$). Discrete FMRP particles cannot be readily detected above the diffuse cytoplasmic signal. Movie consists of four loops; each loop represents 1 min 15 s. Width of region shown is 18 μm.

Movie S3. Behavior of FMRP:GFP in Wild-Type and *BicD* Mutant Chordotonal Organ Neurons In Situ

Chordotonal organ neuronal cluster in filleted third instar larvae expressing UAS-FMRP::GFP (under C155-GAL4 control) in the wild-type and *BicD* mutant background. Dendrites and axons are above and below the cell body, respectively. Although most of the particles are stationary, it is possible to observe directed movement of some particles (arrows), which are less extensive in *BicD* mutants. See main text Figures 3E and 3F for quantification from multiple cells. The low frequency of movement of FMRP puncta even in wild-type cells is consistent with observations of neuronal mRNPs in other studies [11, 19, 20]. Movies consist of four loops; each loop represents 2 min 55 s (left) and 2 min 15 s (right). Width of region shown is 51.3 μm and 36.6 μm for WT and *BicD* mutant, respectively. N.B. reduced levels of FMRP::GFP signal overall in *BicD* mutant neurons, reflecting reduced amounts of total protein in nervous tissue. These movies are of projections of four z-sections of ~1 μm each. Individual z-slices were used for the quantitative data in main text Figures 3E and 3F, permitting more rapid imaging of particles. Fluorescence outside of neurons in the wild-type results from autofluorescence in oenocytes. The oenocytes are visible in a subset of projections of both wild-type and mutant preparations, because of variability in fillet dissection and mounting.
