# Supplementary figures and images for: Bicaudal-D Regulates Fragile X Mental Retardation Protein Levels, Motility, and Function during Neuronal Morphogenesis
Source: Curr Biol. 2010 Aug 24;20(16):1487–92. doi: 10.1016/j.cub.2010.07.016 (PMC2927779; doi:10.1016/j.cub.2010.07.016)

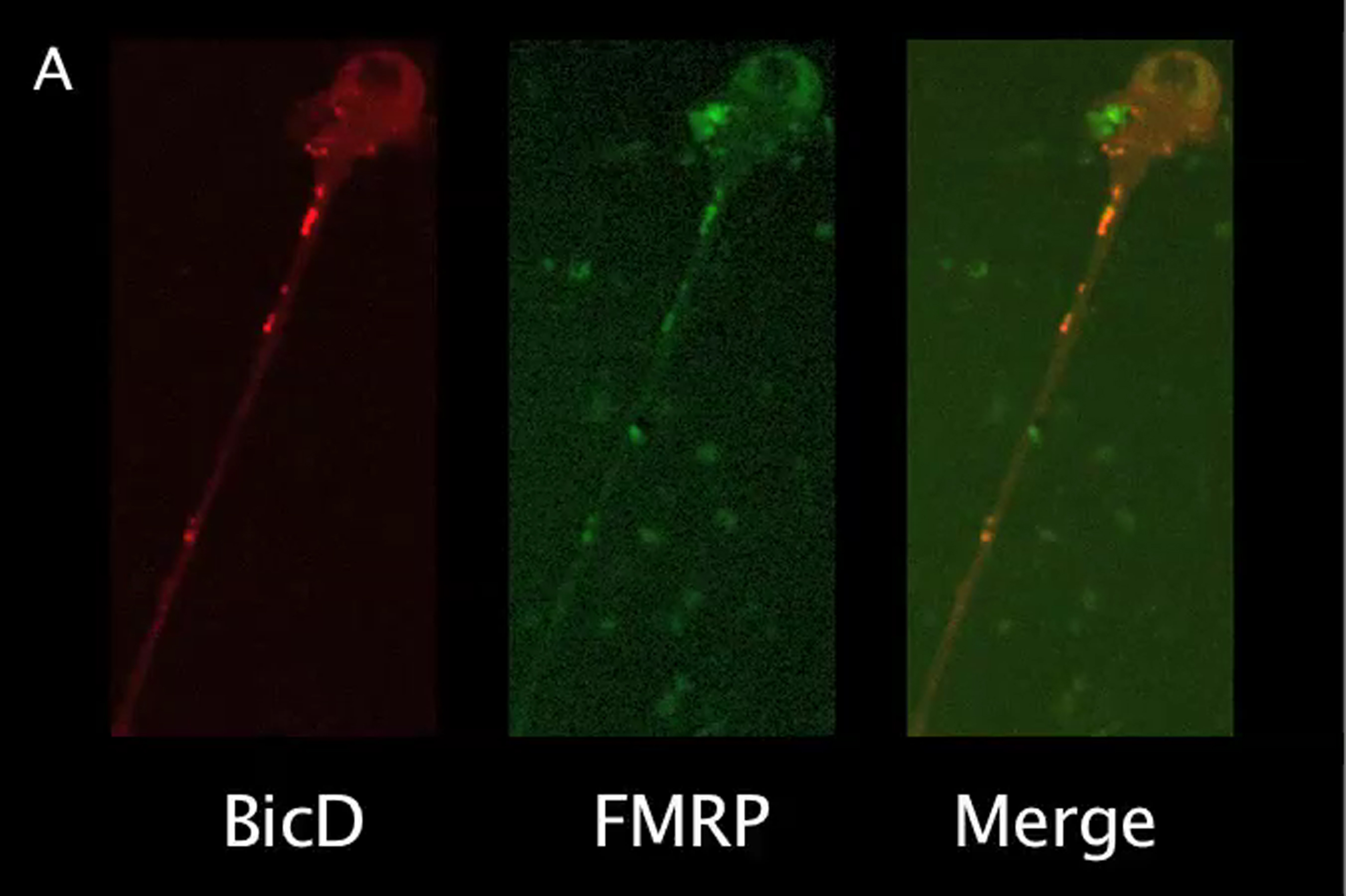

Supplement: Movie S1. Cotransport of BicD::mCherry and FMRP::GFP in Cultured Primary Drosophila Neurons — Consisting of two concatenated movies, A and B. (A) Primary cultured neuron from third instar larvae expressing UAS-FMRP::GFP (green; under C155-GAL4 control) and tub-BicD::Cherry (red). Extensive colocalization of BicD and FMRP in moving particles can be observed. Fluorescence outside the cell comes from debris in the culture medium. Movie consists of four loops; each loop represents 4 min 40 s. (B) Close-up of one particle (arrow), showing correspondence of the BicD and FMRP signals during movement in both directions. Movie consists of four loops; each loop represents 3 min 54 s. [file mmc2.jpg]

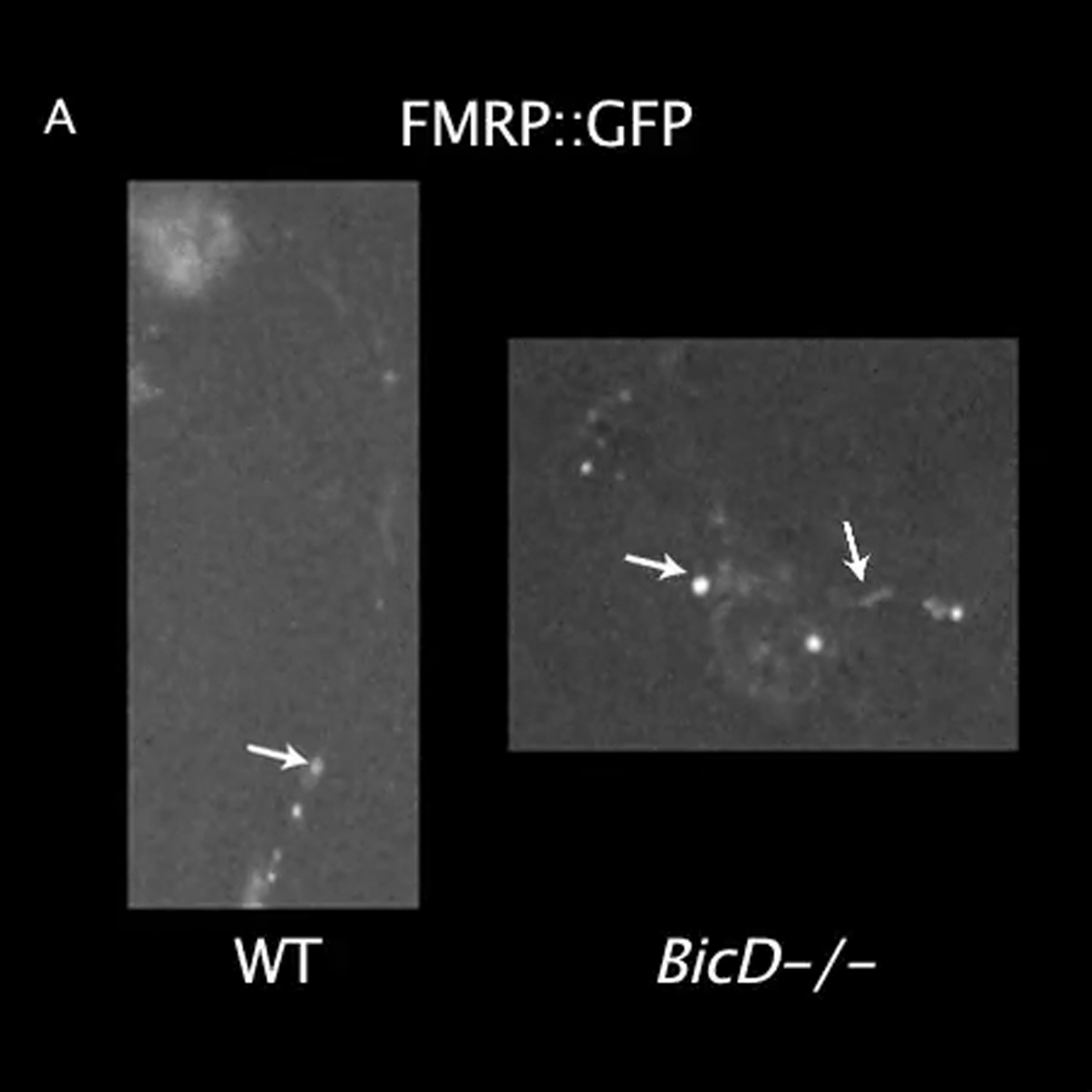

Supplement: Movie S2. Behavior of FMRP:GFP in Wild-Type, BicD, and Khc Mutant Cultured Primary Drosophila Neurons — Consisting of two concatenated movies, A and B. (A) (Left) Primary cultured neuron from a third instar larva expressing UAS-FMRP::GFP (under C155-GAL4 control) in the wild-type background. Some FMRP particles (e.g., arrow) can be seen moving bidirectionally over long distances. (Right) Primary cultured neuron from a third instar larva expressing UAS-FMRP::GFP (under C155-GAL4 control) in the BicD mutant background. Some FMRP particles (arrows) are motile but they mostly perform relatively short, oscillatory movements. In this and other movies BicD mutant genotype is r5/Df119. Movies consist of four loops; each loop represents 2 min 10 s (left) and 2 min 8 s (right). Width of region shown is 14.7 μm and 24.6 μm for wild-type and BicD mutant, respectively. There are higher levels of FMRP::GFP signal in the cell body of the wild-type neuron (top left of image) than in the cell body of the BicD mutant neuron (flanked by arrows) at the onset of filming (i.e., before photobleaching). This reflects reduced amounts of total protein in nervous tissue (Figure 2B). (B) Primary cultured neuron from a third instar larva expressing UAS-FMRP::GFP (under C155-GAL4 control) in a kinesin-1 heavy chain strong hypomorphic background (Khc17/27). Discrete FMRP particles cannot be readily detected above the diffuse cytoplasmic signal. Movie consists of four loops; each loop represents 1 min 15 s. Width of region shown is 18 μm. [file mmc3.jpg]

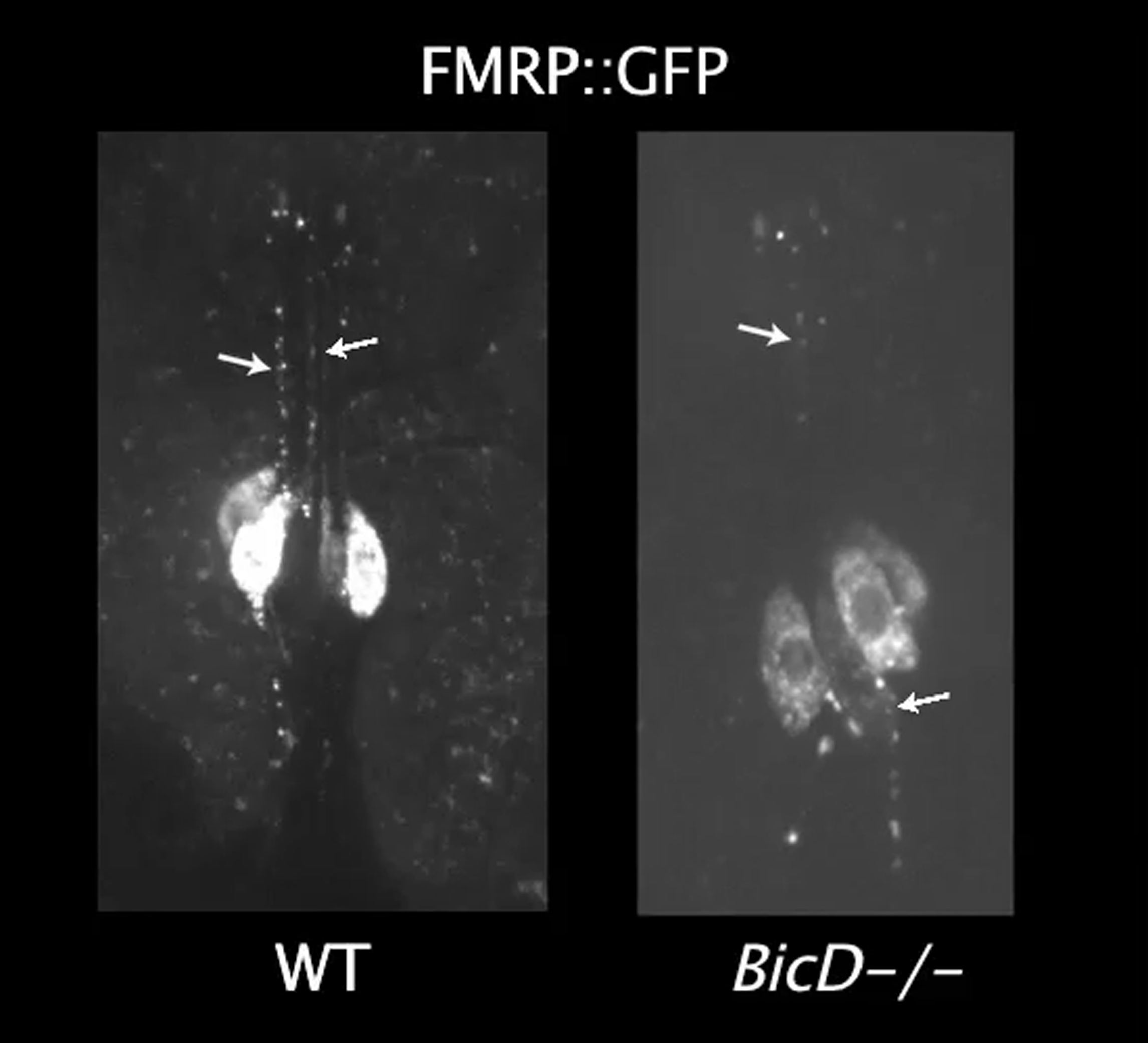

Supplement: Movie S3. Behavior of FMRP:GFP in Wild-Type and BicD Mutant Chordotonal Organ Neurons In Situ — Chordotonal organ neuronal cluster in filleted third instar larvae expressing UAS-FMRP::GFP (under C155-GAL4 control) in the wild-type and BicD mutant background. Dendrites and axons are above and below the cell body, respectively. Although most of the particles are stationary, it is possible to observe directed movement of some particles (arrows), which are less extensive in BicD mutants. See Figures 3E and 3F for quantification from multiple cells. The low frequency of movement of FMRP puncta even in wild-type cells is consistent with observations of neuronal mRNPs in other studies [7–9]. Movies consist of four loops; each loop represents 2 min 55 s (left) and 2 min 15 s (right). Width of region shown is 51.3 μm and 36.6 μm for WT and BicD mutant, respectively. N.B. reduced levels of FMRP::GFP signal overall in BicD mutant neurons, reflecting reduced amounts of total protein in nervous tissue. These movies are of projections of four z-sections of ∼1 μm each. Individual z-slices were used for the quantitative data in Figures 3E and 3F, permitting more rapid imaging of particles. Fluorescence outside of neurons in the wild-type results from autofluorescence in oenocytes. The oenocytes are visible in a subset of projections of both wild-type and mutant preparations, because of variability in fillet dissection and mounting. [file mmc4.jpg]
